# Supplementary material for: Comparison of the Serion IgM ELISA and Microscopic Agglutination Test for diagnosis of Leptospira spp. infections in sera from different geographical origins and estimation of Leptospira seroprevalence in the Wiwa indigenous population from Colombia
Source: PLoS Negl Trop Dis. 2022 Jun 6;16(6):e0009876. doi: 10.1371/journal.pntd.0009876 (PMC9223614; doi:10.1371/journal.pntd.0009876)
Supplement: S1 File. Appendix A—Table with characterization of admission and follow-up serum samples from leptospirosis patients. Appendix B—R code for sample size calculation — (DOCX) [file pntd.0009876.s001.docx]

**Appendix A**

**Table 1:** Characterization of admission and follow-up serum samples from patients, suspected to suffer from leptospirosis, confirmed by MAT and in-house ELISA by the OIE and National Collaborating Centre for Reference and Research on Leptospirosis (NLR), Department of Medical Microbiology and Infection Prevention, University of Amsterdam. The follow-up samples were sent to the diagnostic center of the Medical Department at the Swiss Tropical and Public Health Institute (Swiss TPH) to be tested by the Serion ELISA classic Leptospira IgM, Institut Virion\Serion GmbH, EST125M (“ELISA”) in the frame of the evaluation study described in this article.

| **Sample ID** | **Leptospirosis confirmed on days post onset of illness (DPO)** | **Highest MAT titre**  **at confirmation** | **IgM titre of in-house ELISA from NLR at time of confirmation** | **DPO of follow-up sample sent to Swiss TPH and tested there by the “ELISA”** | **Corresponding highest MAT titre of convalescent sample** | **Corresponding IgM titre of convalescent sample (in-house ELISA from NLR)** | **Probable infecting serogroup** |
| --- | --- | --- | --- | --- | --- | --- | --- |
| 1 | 12 | 320 | 1280 | 48 | 320 | 160 | Pomona |
| 2 | 20 | 320 | 160 | 28 | 320 | 640 | Sejroe |
| 3 | 16 | 160 | 160 | 48 | 320 | 40 | Shermani |
| 4 | 7 | 20 | 1280 | 35 | 1280 | 1280 | Icterohaemorrhagiae |
| 5 | 19 | 10240 | 1280 | 33 | 5120 | 160 | Sejroe |
| 6 | 27 | 160 | 160 | 27 | 160 | 160 | Sejroe |
| 7 | 10 | 320 | 2560 | 41 | 160 | 80 | Not classifiable |
| 8 | 17 | 1280 | 2560 | 31 | 2560 | 2560 | Icterohaemorrhagiae |
| 9 | 11 | 640 | 1280 | 31 | 160 | 320 | Not classifiable |
| 10 | 20 | 160 | 640 | 27 | 640 | 1280 | Bataviae |
| 11 | 4 | 1280 | 1280 | 50 | 5120 | 640 | Icterohaemorrhagiae |
| 12 | 9 | 2560 | 10240 | 50 | 640 | 640 | Icterohaemorrhagiae |
| 13 | 11 | 5120 | 5120 | 22 | 1280 | 5120 | Icterohaemorrhagiae |
| 14 | 16 | 320 | 2560 | 27 | 320 | 640 | Not classifiable |

DPO = Days Post Onset of symptoms; MAT = Microscopic Agglutination Test; OIE = World Organisation for Animal Health

**Appendix B**

R code to determine sensitivity and specificity, applying the method described by Hajian-Tilaki [32] using the (epiR) package in R [33]

library(epiR)

epi.ssdxsesp(test = 0.99, type = "se", Py = 0.4, epsilon = 0.07,error = "absolute", nfractional = FALSE, conf.level = 0.95)

epi.ssdxsesp(test = 0.99, type = "sp", Py = 0.4, epsilon = 0.07,error = "absolute", nfractional = FALSE, conf.level = 0.95)
