# Supplementary material for: Comparison of the Serion IgM ELISA and Microscopic Agglutination Test for diagnosis of Leptospira spp. infections in sera from different geographical origins and estimation of Leptospira seroprevalence in the Wiwa indigenous population from Colombia
Source: PLoS Negl Trop Dis. 2022 Jun 6;16(6):e0009876. doi: 10.1371/journal.pntd.0009876 (PMC9223614; doi:10.1371/journal.pntd.0009876)
Supplement: Attachment — Submitted filename: 220204_ReviewLeptoELISA_ResponsetoReviewers.docx [file pntd.0009876.s002.docx]

**Reviewer's and author`s responses**
 *General comment authors: Thank you very much for taking your time to read our article and for those useful and very justified comments. Moreover, thank you for the polite tone.* *We hope we could respond to your questions.*

**Reviewer #1:** The authors described the study objectives clearly. The study design, including sample size, population sources, and statistical analysis, were explained well. However, the approvals of the Human Research Ethics committee on using serum samples from Group A and B are not described. And I need a rationale on why the study using Bhutanese specimens was granted by the Human Research Ethics committee of the University of Newcastle, Australia, only.

*Comment by authors: Thank you for the friendly introduction. Given that the Bhutanese data is already published, we decided to remove it from the publication. Bhutanese findings can as well be shown and discussed in the discussion. Since it seemed a bit difficult to get a grasp of all the different objectives, we think it is better to simplify the objectives.*

*The sera from patients in the Netherlands (group A) was exempted from ethical review of human subject research by the Medical Ethical Review Committee of the Academic Medical Centre, University of Amsterdam (protocol W12_075#12.17.0092). All data have been de-identified and were not attributable to individual patients. For patient sera with a confirmed diagnosis of a viral, bacterial or parasitic infection (group C) a permis-sion to use the sera for test evaluations was obtained (“Unbedenklichkeitsbescheinigung”: “UBE-15/22”) from the Swiss ethics committee “Ethik Kommission Nordwest- und Zentralschweiz” (EKNZ). According to the EKNZ, based on the Swiss law on science in humans (“Humanforschungsgesetz”), the use of anonymized, bio banked sera (as the blood donor sera, group B) does not require an ethics agreement for the development and validation of diagnostic tests.*

**Reviewer #2:**
1.- Objectives can be clearly summarized and stated. Lines 148-162 may be better included in the materials and methods
*Comment by authors: dear reviewer 2: first I had written the objectives in a short summary. However, I encountered the problem, that readers were confused, what the different study populations were for. Once I summarized it at the beginning, it became clearer. I would hence love to keep the summary at the beginning, so that readers have already a clear image in their head, what we are going to do. I know that strictly speaking, there are elements of the methods section in there. But then we may sometimes have to breach the rules a little bit, if it helps to clarify? I changed the wording, so every line is an objective. I hope it is better this way. However, if you still disagree, I suggest this text:*

*“The objectives of our study were to evaluate the sensitivity and specificity of the commercially available Serion ELISA classic Leptospira IgM using the MAT as imperfect reference test with patient sera from confirmed leptospirosis cases, blood donor sera, with sera from patients with other infections than leptospirosis (all European) and sera from a cross-sectional study in the indigenous population called “Wiwas” of the Sierra Nevada de Santa Marta, North-east of Colombia. In addition, we aimed at estimating the overall Leptospira spp. seroprevalence in the Wiwas indigenous tribe in Colombia.”*

*Thanks for your feedback.*

***Material and methods***

2.- Line 181, indicate in the first time what “TPH” means

*Comment by authors: yes, done, thanks for the observation.*

3.- Line 295. What is an ambiguous result? Why an ambiguous result is considered negative? There were duplicates that could be made to disambiguate

*Comment by authors:* *Unfortunately, this ELISA does not have a clear cut-off for positive and negative results. There is a grey zone, where results could be positive or negative, these are ambiguous. So, we we classified ambiguous results as being negative, as we already had the experience from the Bhutanese data, that the test has many false positive results. I removed the word ambiguous from the text. We did retest duplicates: the inter-assay coefficient of variance over eight measurements from different days and two different samples was 8% and 10% respectively with a standard deviation of 0.1 and 0.11.*

**Reviewer #3:** inclusion of all groups is not clear according with objective of study: compare two diagnoses test and the seroprevalence of Wiwa population.

*Comment authors: we rephrased the objectives and the description of samples in the methods part. Please read again the objectives at the end of the introduction and the beginning of methods. Is it clearer now?*

Two different topics. Sample size should be different: test for a test or population survey.
Ethical OK

*Comment by authors: we added a sample size calculation for the performance of the ELISA for the different settings*

***Results***

**Reviewer #1:** The authors demonstrate results and the data analysis pretty clearly and entirely as the study objectives. The Figures and Tables are tidy and easy to read. In Table 4, the "N" for Group E should be 156 instead of 321. To fulfil more on the study objectives, the accuracy of the "Serion ELISA classic Leptospira IgM" test inferring seroprevalence in sera Group D and E--the endemic areas. Additional positive predictive value (PPV) and negative predictive value (NPV) of the ELISA test in Table 4 (MAT used the reference) will directly point out the failure of using ELISA to investigate the seroprevalence in the endemic areas.
*Comment by author: thank you very much for the good suggestions, which we have followed (see table 4)*

**Reviewer #2:** results
4.- In the table 4, groups A + B should have a single value of "N" (34) to maintain congruence with the other data in the table.

*Comment by authors: thank you, since Reviewer 3 asked to add ELISA and MAT test results to Table 4, we instead separated group A and B and hence kept the total separated. Otherwise, if the table had remained the same, your suggestion would have been correct.*

5.- In group C, why can't the 95% CI for specificity be calculated?

*Comment by authors: it can and we did, merci!*

6.- In group E, the reference test was carried out in 156 people and not in 321.
*Comment by authors: thank you, you are correct. We changed it.*

7.-Line 359. In this section, authors should not incorporate sensitivity in the interpretation, because in group C only specificity is evaluated.

*Comment by authors: this section summarized the findings for the groups A-C. We divided them now by subheading and hope it is clearer.*

8.-Figure 1. Were some of the random samples considered MAT (+)? If any were, it would not be correct to include all the "ELISA negative" (476 or 163) in the sero-negative group, because in theory the ELISA-negative have 2X or 5X with respect to the random sample and if they have some MAT + in the random sample group, there would also be some MAT + in the "ELISA negative" group. With this, the sero-prevalence could be re-calculated.
*Comment by author: All ELISA negative samples of the Colombian Wiwa study population, which were tested by MAT were confirmed as MAT negative. The calculation of the sensitivity and specificity of the ELISA was based on these results.*

*For the sero-prevalence calculation of the Wiwa population, we assumed that all ELISA negative results were negative, whether these had been confirmed by MAT or not. Since none of the ELISA negative results tested positive in the MAT, we decided we could assume that those, which had not been tested by MAT would also test negative. This hypothesis could be disputed. However, the budget was too low to test all negative samples by MAT. We cannot estimate the seroprevalence in the Wiwa population based only on the samples, which had been confirmed by MAT, as it is a biased sample: we chose all ELISA positives and a random sample of the negative.*

*We adapted the figure 1, hoping it is clearer*

9.-The paragraph on lines 441-451 corresponds to the limitations of the study. They must be sent to the corresponding section.

*Comment by authors: we agree and moved the paragraphs accordingly*

**Reviewer #3:** Table 4 should include total, positives by MAT and ELISA.
the use of the term seroprevalence of the study is not clear because are different populations, different countries and sample size, not methodological correct.
*Comment by authors: We added the results to table 4. It requires a lot of explanations at the bottom of the table, as the screening procedure needed to be explained to understand the numbers. I hope it is comprehensible. I hope Figure 1 explains the procedures better now. Sero-prevalence is only used for the last objective in the study.*

**Conclusions**

**Reviewer #1:** The authors conclude the study reasonably, including all limitations of the analysis and study design.

*Comment by authors: merci, nice to hear ☺*

**Reviewer #2:** 9.- The first paragraph (lines 416-424) is part of the objectives and methodology that has already been explained. It is recommended to start the discussion with the main results obtained.
*Comment by authors: ok, we rephrased* *the first paragraph*

10.- Lines 458-479 support the over and underestimation assumption. Therefore, they must be linked to the sentence that mentions it.
*Comment by authors: we rearranged the sentences*

11.-Line 494. Has the hypothesis of low specificity been evidenced in other studies of leptospira or other spirochetes? If so, include those studies as references.

*Comment by authors: We could not find any. Do you want me to remove the hypothesis?*

**Reviewer #3:** It is inconsistent with the introduction the profile of risk of Wiwa population and the conclusion presented.

*Comment by authors: Yes, we were also astonished about the low seroprevalence in the Wiwa population. Our hypothesis was to find a high prevalence, based on the description of the Wiwa`s living conditions described in the introduction. But we cannot change the introduction if our hypothesis was refuted. We mention in the discussion that results do not match what we expect, based on the results in the literature and their living conditions.*

The statement "ELISA is technically working well..." should include the concrete arguments for that.

*Comment by authors: we removed “technically working well”*

The discrepancy between MAT and ELISA should be analice from the theory more than make comparisons with results from other species.
*Comment by authors: we apologize, but we are not quite sure what you mean with “inferring from theory”. Given that leptospirosis is a zoonosis, it is -in our opinion- interesting to see the burden in other species, which may transmit leptospires directly or indirectly.*
conclusions about seroprevalence in Wiwa population should be discuss and evaluate in a future with comorbilities from the region as other febrile syndromes from infectious diseases.

*Comment by authors: we agree and we added a sentence.*

--------------------

Editorial and Data Presentation Modifications?
Use this section for editorial suggestions as well as relatively minor modifications of existing data that would enhance clarity. If the only modifications needed are minor and/or editorial, you may wish to recommend “Minor Revision” or “Accept”.

**Reviewer #1:** I suggest a Minor Revision for this manuscript. My significant comments are as follows:
1. show IRB approval for study Group A & B and IRB multi-centre or Bhutanese IRB approval for study Group D
2. correct the "N" number in Table 4 for Group E
3. add PPV (95%CI) and NPV (95%PI) in Table 4 for Group D and E.

**Reviewer #2**: Be congruent with the number of decimal places to include in the percentages.

In Abstract: Maintain the same units at the conclusion of the% seroprevalence (line 59)

**Reviewer #3:** Maybe a figure (a different figure than a diagram) to show some results will be more easy to understand (388-399).

--------------------

**Summary and General Comments**
Use  this section to provide overall comments, discuss strengths/weaknesses of the study, novelty, significance, general execution and scholarship. You may also include additional comments for the author, including concerns about dual publication, research ethics, or publication ethics. If requesting major revision, please articulate the new experiments that are needed.

**Reviewer #1**: Some significant results were published in Pathogens; the whole story is still beneficial for Leptospira diagnosis or seroprevalence study. It emphasizes the essential test validation before use in disease-endemic areas.

**Reviewer #2**: This study has the strength of having investigated the performance of a serological test for leptospirosis in a non-endemic setting (after onset of disease) and in an endemic setting where further research is still necessary.

**Reviewer #3:** I think that more importance should be done to wiwa results. **And as secondary results should be comparisons with other populations.** That implies more emphases at those results and to begin with Wiwa populations context, methodology and results.

*Comment authors: dear Reviewer, unfortunately, the editors asked from us exactly the opposite, making the statement: “The goal of estimating sero-prevalence in the Colombian study population is a secondary outcome of the accuracy study, and stated, most of the report should be focused on the diagnostic test evaluation study following recommended guidelines.”. In light of these comments, we hope you can accept -without taking offense-, that we did not follow this recommendation of yours. In hindsight, we could have probably published the Wiwa seroprevalence data on its own as a short report, but no/very low prevalence is always more difficult to publish and there would have been a lot of repetition, describing the diagnostic tests.*

--------------------
