# Supplementary material for: Comparison of the Serion IgM ELISA and Microscopic Agglutination Test for diagnosis of Leptospira spp. infections in sera from different geographical origins and estimation of Leptospira seroprevalence in the Wiwa indigenous population from Colombia
Source: PLoS Negl Trop Dis. 2022 Jun 6;16(6):e0009876. doi: 10.1371/journal.pntd.0009876 (PMC9223614; doi:10.1371/journal.pntd.0009876)
Supplement: Attachment — Submitted filename: 220411_Review2_reply_author.docx [file pntd.0009876.s003.docx]

**Reviewer's Responses to Questions and author replies**

***1.*** *<b>Key Review Criteria Required for Acceptance?</b></br></br>
As you describe the new analyses required for acceptance, please consider the following:</br></br>

<b>Methods</b></br></br>
-Are the objectives of the study clearly articulated with a clear testable hypothesis stated?</br>
-Is the study design appropriate to address the stated objectives?</br>
-Is the population clearly described and appropriate for the hypothesis being tested?</br>
-Is the sample size sufficient to ensure adequate power to address the hypothesis being tested?</br>
-Were correct statistical analysis used to support conclusions?</br>
-Are there concerns about ethical or regulatory requirements being met?</br></br>*

**Reviewer #1:** Authors describe the objectives of each experiment clearly. The primary aims are evaluating the analysis sensitivity and specificity of the Serion ELISA classic Leptospira IgM using a) 14 confirmed leptospirosis cases originated from Netherland, b) 20 healthy blood donors, c) 41 confirmed other infections originated from Switzerland, d) 321/489 sera from volunteers (healthy or ill) originated from Colombia.

The objectives of the group d investigation (line 191-193) are arguable. In basic immunology, IgG antibody screenings are usually performed for investigating seroprevalence. The IgM ELISA was used for seroprevalence of leptospirosis in this study. Authors should describe reasons for use this unexpected study design. Previous studies on validating IgM and IgG for Leptospira seroprevalence in comparison are recommended in the Introduction section.  Emphasizing using MAT on single sera for the seroprevalence (not diagnosis) as a reference method should be done to remind readers.

*Reply author: IgM class antibodies usually appear earlier than IgG class antibodies, and remain for months or years at a low detectable titre after Leptospira infection. IgG antibodies may not be detected at all, or for only a short period, or persist for several years [4, 15]. Therefore, IgM ELISA may be a better test to detect Leptospira antibodies even in a cross-sectional study. I mention this now in the introduction. Previous studies using IgM are mentioned in the discussion. I prefer to leave them in the discussion. I added a section on MAT use in seroprevalence studies.*

The study design of the seroprevalence in group d is questionable. There is a cross-sectional study design that perform a reference test first and then performing case-control sampling (based on the reference test results) to further perform index test (the ELISA test in this study). Authors should describe the type of the cross-sectional designs selected in this study and the reason of selections.

*Reply author: Apologies, but I do not understand the critique and what I should change. I know many studies, which use an ELISA as screening test and the reference test as confirmatory test. We did not conduct a case-control study. I described the study design of the cross-sectional study, so I am not sure what else to describe.*

PPV and NPV are not needed for group a, b or c.

*Reply author: I deleted PPV and NPV for group a, b or c. (Note to editors: the previous reviewer had asked me to add them, they were not there in my first version, so now I remove them again)*

In my personal opinion, all a,b, c and d should be done for only one primary aim: to investigating the accuracy of the ELISA test in survey sero-prevalence (both past and current infection) compared to MAT using single sera collected during leptospiremia, leptospirurea, and no Leptospira in bodies.

*Reply author: apologies, I am not sure I understand the comment. Do I understand correctly, that you suggest deleting the estimation of the sero-prevalence in the Wiwa population? If this is the case, I would ask the PLOS editors to take this final decision, as three other reviewers did not have this opinion and I would find it a pity to lose this part of the results.*

**Reviewer #2: objectives.**
Please to include the text suggested for the author:
"“The objectives of our study were to evaluate the sensitivity and specificity of the commercially
available Serion ELISA classic Leptospira IgM using the MAT as imperfect reference test with patient
sera from confirmed leptospirosis cases, blood donor sera, with sera from patients with other
infections than leptospirosis (all European) and sera from a cross-sectional study in the indigenous
population called “Wiwas” of the Sierra Nevada de Santa Marta, North-east of Colombia..."

*Reply author: I have changed the objectives as requested*

**Reviewer #3:** (No Response)

--------------------

***2.*** *<b>Results</b></br></br>
-Does the analysis presented match the analysis plan?</br>
-Are the results clearly and completely presented?</br>
-Are the figures (Tables, Images) of sufficient quality for clarity?</br></br>*

**Reviewer #1:** Figure 1--It is not correct to pool ELISA and MAT results (called sero-positive and sero-negative).
Table 4 --the 124/32 (instead of NA) should be add in the ELISA (pos/neg) column in row D ii

*Reply author: I have changed the figure and table input as requested*

Estimating sero-prevalence based on the ELISA in Wiwa population were less accuracy as the significant low specificity compared to those estimated by MAT. Combination results from samples having ELISA results only might be another kind of bias.

*Reply author: apologies, I do not understand the comment (English!) and therefore do not know which changes are required.*

**Reviewer #2:** Table 4.
specificity values of Group B are incorrectly added within the group A row. (Group A are cases, there are no "negative samples", so it should not have any specificity value)

*Reply author: yes, you are correct, the data was not in the correct row. I moved it down.*

In group C, please indicate what the value of 100% specificity corresponds to. At 3 MAT-/ELISA- ? if so, clarify it in the legend below

*Reply author: it is a specificity, which is not calculated from MAT results, but from diagnostic test results being negative for other pathogens. I mention this now below the table, thank you.*

**Reviewer #3:** (No Response)

--------------------

***3.*** *<b>Conclusions</b></br></br>
-Are the conclusions supported by the data presented?</br>
-Are the limitations of analysis clearly described?</br>
-Do the authors discuss how these data can be helpful to advance our understanding of the topic under study?</br>
-Is public health relevance addressed?</br></br>*

**Reviewer #1:** Authors conclude that the IgM ELISA test was less accuracy when use for Leptospira seroprevalence compared to MAT. Authors argue on the less sensitivity of MAT (single sera) when use as a diagnosis tool (line 479-483). However, I did not see the relevant to the group d experiment.
Even the results clearly show low accuracy of the IgM ELISA test, authors insist that ".. IgM detection test can be useful in screening for Leptospira exposure in the recent past.." (510-517) based on a citation of book reference. Serious discussion with previous reports on this issue are lacking.

*Reply author: Again my apologies, I do not understand the comment very well. I now changed the sentence on the performance of this ELISA for cross-sectional studies: “the ELISA is not recommended, due to its low specificity”.*

**Reviewer #2**: Discussion: About your hypothesis in lines 422-424, It is Ok after your feedback.

**Reviewer #3:** (No Response)

--------------------

***4****. <b>Editorial and Data Presentation Modifications?</b></br><br/>
Use  this section for editorial suggestions as well as relatively minor modifications of existing data that would enhance clarity. If the only modifications needed are minor and/or editorial, you may wish to recommend “Minor Revision” or “Accept”.*

**Reviewer #1:** (No Response)

**Reviewer #2:** none

**Reviewer #3:** 5 In the title could be more accurate to use leptospirosis seroprevalence

51 Sensitivity value at cross sectional study in Colombia is missing in the abstract

*Reply author: thanks for spotting this. I added the sensitivity.*

184 “most likely seronegative for Leptospira spp..” could means doubt about leptospirosis diagnoses and cannot be objective to evaluate cross reaction.

*Reply author: I now changed the sentence on the performance of this ELISA for cross-sectional studies: “the ELISA is not recommended, due to its low specificity”.*

254 Should be interesting pointed the reason to use OIE standards for human test, and not WHO.

*Reply author: I am a vet and the guidelines are useful for the MAT in humans and animals. But I can change the reference to WHO guidelines, if this is deemed necessary.*

278 If you referred to “endemic places” to Colombia, should be better to describe it explicitly, because its methodology of the study.

*Reply author: apologies, I am not sure I understand your request. I removed “endemic”.*

--------------------

***5.*** *<b>Summary and General Comments</b></br></br>
Use this section to provide overall comments, discuss strengths/weaknesses of the study, novelty, significance, general execution and scholarship. You may also include additional comments for the author, including concerns about dual publication, research ethics, or publication ethics. If requesting major revision, please articulate the new experiments that are needed.*

**Reviewer #1:** I concerns on the objectives and study designs used in this study. It might be better that authors present a major and accuracy key message to readers with evidence based style. The manuscripts can be confusing as the ELISA was evaluated as a diagnosis tool and a sero-prevalence in the same article. As a reader of PLoS NTD, I eager to know if IgM ELISA (using non-pathogen Leptospira antigen) is useful for seroprevalence in the low income countries with leptospirosis endemic areas or not.

*Reply author: I do not understand your comment about the “evidence-based” style. Our results are based on valid study designs and diagnostic test results, which are analysed with recognized statistical tools. Further, I tried to find evidence in the literature, which is cited in the introduction and the discussion.. However, not everything we conclude can be based on previous literature, otherwise we will never advance with new ideas.*

*If you suggest removing the part on sero-prevalence, I ask the editors to take this decision, as the other three reviewers did not suggest it. One reviewer even thought that the sero-prevalence study was very interesting and should get more attention. So, I will wait for a final verdict by the editors. Thank you.*

I am confused by Abstract. And please check the information accuracy in the abstract as well.

*Reply author: I rephrased the abstract. I hope it is less confusing.*

**Reviewer #2:** none

**Reviewer #3:** (No Response)

--------------------

PLOS authors have the option to publish the peer review history of their article (<a href="<https://journals.plos.org/plosntds/s/editorial-and-peer-review-process#loc-peer-review-history>" target="_blank">what does this mean?</a>). If published, this will include your full peer review and any attached files.<br><br>

If you choose “no”, your identity will remain anonymous but your review may still be made public.<br><br>

<b>Do you want your identity to be public for this peer review?</b> For information about this choice, including consent withdrawal, please see our <a href="<https://www.plos.org/privacy-policy>" target="_blank">Privacy Policy</a>.

Reviewer #1: No

Reviewer #2: Yes: Antonio Flores

Reviewer #3: Yes: Janeth Perez-Garcia

*Reply author: thank you for taking the time to review and comment this manuscript*
